# Making the case for strong health information systems during a pandemic and beyond

**DOI:** 10.1186/s13690-021-00531-5

**Published:** 2021-01-29

**Authors:** Andrea E. Schmidt, Linda A. Abboud, Petronille Bogaert

**Affiliations:** 1 Austrian Public Health Institute (Gesundheit Österreich), Department of Health Economics and Health Systems Analysis, Stubenring 6, 1010 Vienna, Austria; 2 Department of Epidemiology and Public Health, Sciensano, Brussels, Belgium; 3 School of Social and Behavioral Sciences, Tilburg University, Tilburg, the Netherlands

**Keywords:** Health information systems, Capacity-building, COVID-19, Vulnerable groups, Data linkage, Infrastructure

## Abstract

**Background:**

The Sars-CoV-2 pandemic exacerbates existing inequalities across health care systems globally, both within countries and between countries. It also highlights, like no other crisis before, existing weaknesses in health information systems (HIS). This article summarizes these key challenges for HIS in times of a pandemic and beyond, with a focus on European countries. It builds on the experiences of a large consortium representing HIS experts in key positions in national public health or similar institutes across Europe.

**Methods:**

Data were collected in bi-weekly conference calls organized by the InfAct project between February and June 2020. Emerging themes were clustered and analysed around a WHO framework for health information systems (HIS). We analyse strengths of HIS at two levels: (i) dealing with health information directly, and (ii) dealing with other parts of information systems that allow for a holistic assessment of the pandemic (including health-related aspects).

**Results:**

The analysis highlights the need for capacity-building in HIS before a pandemic hits, the relevance of going beyond health information only related to health care but taking a broader perspective (e.g. on vulnerable groups), the need for strong reporting systems on staffing numbers and in primary care. Further, data linkage emerges as a crucial precondition to identify unmet needs for essential health care services in a timely manner. Finally, room for innovation and digitalisation is key to be able to react flexibly in times of crisis. Trust for health information stakeholders is another important factor to create strong HIS.

**Conclusions:**

The strengths and shortcomings of European HIS that have been observed during the COVID-19 crisis highlight the need for strong HIS beyond the crisis. The experiences reported leave as a central message that successful reactions to the pandemic are (also) grounded in strong HIS that ultimately not only benefit the health of the population but also create a number of economic and psycho-social benefits. Strong data reporting schemes may also support fine-tuning of containment measures during a pandemic as well as transition phases.

## Background

The Sars-CoV-2 pandemic exacerbates existing inequalities across health care systems globally, both within countries and between countries. It also highlights, like no other crisis before, existing weaknesses in health information systems (HIS). In fact, HIS are considered as one of the main building blocks of health systems by the World Health Organization (WHO) [1–3]. During this pandemic, certain key components of HIS have gained particular importance. This article summarizes these key challenges for HIS in times of a pandemic and beyond, with a focus on European countries. It builds on the experiences of a large consortium representing HIS experts in key positions in national public health or similar institutes across Europe. The main struggles identified in times of a pandemic are in line with what has been observed in prior time as common weaknesses of HIS [4], as showcased by the ongoing works of the Joint Action on Health Information- InfAct,^1^ but also display some particularities.

We argue that HIS need to be strong ideally before a crisis hits, which means that constant investments are required for an efficiently functioning HIS. Strong HIS allow for a coordinated response in times of public health crisis and thus implicitly bear a large potential for overall economic and social benefits (cf. [5]). Challenges identified should be dealt with in a systematic way for the sake of population health, as well as for the sake of economic and social objectives. The next section summarizes the data and methods used for the discussion presented here. We continue with a presentation of the conceptual approach used, and finally the analysis itself ending with some concluding remarks.

## Methods

Data were collected in bi-weekly conference calls organized by the InfAct project between February and June 2020. The conference calls aimed to identify problems with regard to HIS in the COVID-19 crisis, to facilitate and to exchange good practices for crisis response across Europe. Additionally, InfAct functioned as a rapid response team by connecting key experts to respond to pressing questions. This commentary thus aims to systematize the struggles faced by national public health institutes with regard to health information in the crisis and beyond, based on a qualitative thematic clustering analysis of the themes discussed during these conference calls. In the seven meetings held, a total of 16 countries participated at least once, a total of 12 countries participated in at least half (3 meetings) of the meetings (Table 1).

**Table 1:** Overview of countries that participated in meetings which served as basis for data collection

	Country	Number of meetings joined out of 7
1	Austria	7
2	Belgium	7
3	Croatia	6
4	Czech Republic	6
5	Finland	3
6	France	7
7	Germany	4
8	Italy	1
9	Lithuania	4
10	Malta	3
11	Netherlands	1
12	Poland	2
13	Portugal	7
14	Slovenia	4
15	Spain	7
16	United Kingdom	1

### Framework and conceptual approach

In our analysis, we follow a well-established WHO framework [6] which covers the following domains of HIS: (i) resources, (ii) indicators, (iii) data sources, (iv) data management, (v) national HIS data quality/information products, (vi) dissemination and use. We adapt this framework in order to analyse the following two levels (from weak to strong):

1. Direct health information: Are countries able to process health information directly related to the pandemic within the health care sector (e.g. related to the maintenance and provision of essential health care services)?
2. Holistic health information: In addition, are countries able to process information not directly related to the health care sector (e.g. socio-economic determinants of being affected by Sars-CoV2 infection, or logistics information/information about infrastructure of laboratories with testing capacities), and establish linkages with information described under (1)?

Countries may be strong on one aspect, yet weak on the other, resulting in a matrix-like classification (Table 2). We do not aim to present an empirical analysis of where countries could be placed in the matrix, but rather aim to conceptualise HIS in times of crisis from a theoretical standpoint (based on real-life experiences reported in our data).

**Table 2:** Conceptual approach

		**(1) health information directly related to the pandemic & on essential health care services**	
	Assessment:	**weak**	**strong**
**(2) holistic health information (incl. non-health information)**	**weak**	Countries with weak direct health information, and weak holistic health information	Countries with strong direct health information, but weak holistic health information
	**strong**	Countries with weak direct health information, but strong holistic health information	Countries with strong direct health information, and strong holistic health information
Domains for assessment following [6] in dimensions (1) and (2):		(i) *Resources*: e.g. Which resources for HIS should, ideally, be available (incl. HIS-related legislation)? (ii) *Indicators*: e.g. What kind of information, and in particular, which indicators have proven particularly useful, and who is involved in deciding which indicators are being collected (national and international participation)? (iii) *Data sources*: e.g. Which data sources and data sets (should) exist in a comprehensive manner and be updated regularly? (iv) *Data management*: e.g. Are there state-of-the art protocols for data management? Which aspects of data management need to be considered especially? (v) *HIS information products*: e.g. How could (or should) health information products look like for policy-makers, the media, or researchers? and at what level of disaggregation and/or in which quality? (vi) *Dissemination and use*: e.g. Which forms of interactions with policy-makers, the media and the (inter-)national research community have proven particularly useful?	

In each of these areas we identify HIS related actions based on our analysis. Using the WHO framework as a guidance [6], the next section highlights key challenges for HIS, in times of the pandemic and beyond, for each of these aspects.

### Analysis

#### Resources: ensure capacity-building before the pandemic hits

The analysis of our data shows that HIS grounded on sufficient capacity outside of pandemics are able to expand and create strong surveillance systems during crises. These include as key elements a legal basis for all aspects concerning HIS, an adequate number of health and care staff, and the necessary infrastructure with regard to health care institutions and ICT infrastructure. The experience with the COVID-19 pandemic in European countries shows that capacity-building should take place before rather than during a pandemic. Sustainability in funding of routine data collection mechanisms, automated communication flows between institutions and government levels are key elements, as well as compliance with data protection laws. In this context, governance structures and governance documents (e.g. an HIS strategy or written protocols on patient safety and hygiene) also play a role. The capacity-building refers mainly to direct health information, i.e. dimension (1) of our conceptual framework, but could also extend to the holistic dimension (2) (cf. Table 2). For instance, financing of health information infrastructure and data linkage are a precondition for ensuring sufficient capacity, as is availability of trained legal experts to deal with questions related to data protection. Sufficient resources are a key for faring strongly on the domains mentioned in Table 1 (legislation, staff, and infrastructure) in order to ensure strong HIS in times of crisis or a pandemic.

#### Indicators: collect longitudinal information on vulnerable groups

Monitoring the wider effects of COVID-19 requires longitudinal data from health systems and surveys with reference points from before the time of the pandemic, using validated questions. Building on high-quality data allows a broad understanding both of the drivers of pandemics and the impact of pandemics on health and non-health related aspects. For example, in Belgium, COVID-19 specific health surveys, which can be benchmarked with previous surveys, are organised. The Belgian COVID-19 health surveys help scientists and policymakers to estimate the impact of the COVID-19 crisis on health. They allow mapping trends and studying various public health topics. Attention is given to physical, mental and social well-being, and the use of health services. As another example, the Finnish Institute for Health and Welfare (THL) prepared a rapid impact assessment about the effects of COVID-19 epidemic on the population’s service needs, the service system and the economy. As part of a sero-prevalence study, a short web-based questionnaire was administered to learn more about quality of life, functional capacity, use of health and social care services for non-COVID-19-related health issues (e.g. chronic conditions), smoking or alcohol use, to name a few. In addition to surveys, a participatory process is desirable in which users (e.g. from the research community) are involved in evaluating the usefulness of the indicators collected, and in identifying potential gaps.

For example, and relating to the holistic level of health information (see (2) in Table 2), it has become apparent that data on certain socio-economic groups may be of high relevance, more so during a pandemic. Socially vulnerable groups (e.g. homeless people, drug users, sex workers) need to be monitored closely, as precarious working and living conditions have shown to foster the occurrence of outbreaks of COVID-19 [7, 8]. Administrative data (e.g. registries) that allow for identification of specific social settings or socio-economic characteristics of the population, in addition to health-related characteristics, are particularly valuable in this context. Countries with a health-in-all-policy approach that is reflected in the data collected and reported, are in a better position to monitor the health of the total population with specific attention to the situation of vulnerable groups.

Relating to direct health information (level (1) in Table 2), people in need of long-term care are another vulnerable group in the current pandemic, as a large share of those most at risk of dying from COVID-19 are older people and/or live in care homes [9]. Countries with HIS that integrate information from the health and the long-term care sector are likely better able to respond to the crisis. Furthermore, other vulnerable groups are those with specific health conditions associated with lower socio-economic conditions that may increase the risk for severe COVID-19 disease. For example, people with comorbidities (e.g. obesity) have been shown to be at risk of experiencing severe COVID-19, with prevalence of obesity being significantly elevated in lower socio-economic groups. In combination with a lack of health literacy often associated with lower socio-economic status, people at risk may not always realise they are. It is therefore crucial that HIS collect information on the general population including the people with co-morbidities who are not necessarily seeking medical care for these conditions. In general, countries with a focus on using routine mechanisms for data flow may be in a better position to monitor specific groups, e.g. flows between administrative data and vital statistics, flows across settings such as hospitals, laboratories and care homes.

#### Data sources: establish strong reporting systems on staffing numbers and in primary care

Referring to direct health information (level (1) in Table 2) our analysis shows that countries which have comprehensive and complete reporting systems for staffing numbers in hospitals and care homes may be better able to manage and create separate teams. Equally, going beyond direct health information but more towards a holistic approach (level (2) in Table 2) HIS that systematically collect information on available materials (e.g. in hospitals) including medication and collaborate with other sectors (e.g. trade, economic sector), are in a better position to respond to the crisis, and clarify logistical questions more rapidly than other countries.

As a second point to consider under level (1) relating to direct health information, countries with sufficient capacities in primary health care (incl. Documentation systems) will be better able to monitor suspicious cases for COVID-19 in the phases following the peak. Countries like France or Spain monitor people with COVID-19 symptoms from the onset, including collection of information on time needed between occurrence of first symptoms until testing and/or diagnosis. In addition, where health literacy of primary health care providers is strong, they may be better able to communicate potential risks during the pandemic to their patients, or reach out to patients at risk proactively. Similarly, countries with the respective reporting systems to monitor suspicious cases (also e.g. via inbound telephone hotlines or in primary care) have been able to develop early warning systems (e.g. France, Spain) that may help prevent future waves of the pandemic. Similarly, the capacity to collect mortality data varies across countries depending on the ability to test. This would require logistic capacity and information systems that link mortality reporting from different sources (within countries and internationally).

#### Data management: link data sources to identify unmet needs for essential health care timely

It is likely that during the peak of the crisis, demand for essential health care may have decreased. People may postpone medical care, including the use of preventive services thus making adequate and reliable data management a crucial element. For instance, a crowding out of essential, regular health services is likely to have taken place in many countries (see e.g. [10, 11]). Relating to strengthening direct health information (level (1) in Table 2), foundations for excellent data management procedures need to be laid before the outbreak of a pandemic. Trust relationships with data providers need to already be established, making data providers more likely to be engaged and, for instance, ready to put in the extra time during a crisis. Also, data managers will already be comfortable with the software tools in use. Investing in training staff on a regular bases before a crisis, will ensure that staff can process data more easily when a crisis hits.

Countries with strong HIS (direct health information, cf. level (1) in Table 1) allow for detailed reporting on health care use in all settings, including ambulatory care, and reporting on continuity of care between primary and secondary care. These are likely to be in a better position to build up capacities for maintaining and managing regular health services (not directly related to the pandemic) during a crisis. WHO recommends an “expanded (dual) dashboard of service coverage and delivery indicators and the use of key tracer indicators on utilization patterns and mortality on both COVID-19 and non-COVID-19 conditions to manage a dual-track health system” (WHO, 2020:8). In the Netherlands, foresight studies are conducted, in which scenarios are developed to provide insight into longer-term health impacts, accounting for the current effect of the pandemic. Also, in the Czech Republic, a comprehensive COVID-19 monitoring system has been established, including information on COVID-19 confirmed cases, their medical history, general inpatient / intensive care, and outcome, ultimately allowing to monitor not only status of the epidemic, but also potential long-term health outcomes of recovered COVID-19 patients. In general, coordination of data collection across sectors, e.g. via a unique identifier or a centralised data storage platform for all health care sectors may be a crucial element in handling the pandemic, as it is done for instance in Denmark or Norway. In addition, data quality checks are important too, e.g. when it comes to doing cross-checks and taking out duplicates, thus generating reliable statistics (e.g. on COVID-19-cause mortality).

#### Information products: introduce room for innovation and digitalisation

According to the country experiences in our data, having the legal basis clarified for the use of video or phone consultations beforehand enabled some countries to rapidly reduce human contacts between patients and doctors substantially during the pandemic. Also, countries where innovation - in both direct health information systems and those not directly linked to health - is incentivised, specifically funded or promoted, were better able to develop flexible HIS to deal with the crisis (incl. Telemedicine solutions). As an example, Austria had laid the foundations for e-prescriptions already before the crisis and was therefore able to facilitate implementation during the pandemic.

The crisis has also shown the potential for the use of mobile services and digital technologies in the health sector, as well as difficulties in harmonised data protection legalisation across EU countries. In fact, many countries have seen limited use e.g. of contact tracing apps, as they did not comply with the EU’s general data protection regulation (GDPR). Coordinated efforts at EU level may bear some potential in this respect, e.g. an app developed by the EC (DG SANTE). Contact tracing may however be context-specific and bear limited success in European countries as compared for instance to Asian countries. Also, non-digital solutions may be found that can be highly innovative. For example, in Wales, a collaboration between academia public health agency, health service and the Government was created to respond to the COVID19 challenge and provide intelligence to guide policy and practice interventions to minimise direct and indirect harms to the population.

#### Dissemination and use: create public trust for health information

The WHO (2020) recommends “simple , timely, effective, evidence-based and honest communication, transparency to build public trust, manage infodemics”. Transparent dissemination of data on health care utilisation (e.g. using dashboards) tends to create public trust. Good practice examples in this respect are Portugal and France. As the WHO recommends, data analytics on public health decisions must be data driven at all times. WHO also recommends “to monitor the mental and physical impact of various measures on the population, as well as the public’s willingness and ability to adhere to those measures.” WHO particularly focuses on countries with public policy levers outside the health sector and HiAp approaches [12].

This pandemic has shown that reporting on mortality numbers varies widely across European countries, and sometimes even at subnational level. It is therefore required that a strong HIS communicates in a clear and transparent language. This includes for example communicating on complex differences in reporting methodologies, transparent information on how numbers are being produced, and also the transparency with which evidence supporting re-opening after lockdown is communicated. Important questions for evaluating the HIS in this respect are: How does the government interact with experts in the field? How do experts report evidence to policy-makers? Are evidence-building documents available to the general public? How does interaction with public opinion take place as a consequence of measures taken (e.g. social media, press conferences)?

## Conclusions

The strengths and shortcomings of European HIS that have been observed during the COVID-19 crisis highlight the need for strong HIS beyond the crisis [13]. In other words, the experiences reported in our commentary leave as a central message that successful reactions to the pandemic are (also) grounded in strong HIS that ultimately not only benefit the health of the population but also create a number of economic and psycho-social benefits, as strong data reporting schemes may support fine-tuning of containment measures during a pandemic as well as transition phases.

The findings presented here are also supported by HIS assessments carried out in the Joint Action for Health Information, called InfAct. InfAct is a 36 months project, includes 40 partners in 28 EU and associated countries. Through country collaboration, InfAct streamlines health information activities across Europe. It builds towards a sustainable and solid infrastructure for EU population health information and strengthens its core elements based on capacity building, health information tools and political support. The HIS assessments within InfAct Work Package 5 (Status of health information systems in MS and regions) were carried out during 2019 in 9 EU countries. This task was carried out using an adjusted version of the WHO health information system assessment tool, and aimed to address health information inequalities between countries through a peer-review exercise, and support the participating countries in identifying action points for direct and long-term improvement and strengthening of their HIS. The process and results of the assessments are currently being compiled and processed and will be published.

## Data Availability

The information analysed during the current study are not publicly available due confidentiality but are available from the corresponding author on reasonable request.

## Abbreviations

WHO: World Health Organization
HIS: Health information system

## Footnotes

1 Website see https://www.inf-act.eu/ (accessed on 2nd August 2020).
